# Transcriptomic Profiling of Paired Primary Tumors and CNS Metastases in Breast Cancer Reveals Immune Modulation Signatures

**DOI:** 10.3390/ijms26146944

**Published:** 2025-07-19

**Authors:** Ana Julia Aguiar de Freitas, Muriele Bertagna Varuzza, Stéphanie Calfa, Rhafaela Lima Causin, Vinicius Duval da Silva, Cristiano de Pádua Souza, Márcia Maria Chiquitelli Marques

**Affiliations:** 1 Molecular Oncology Research Center, Teaching and Research Institute, Barretos Cancer Hospital, Barretos 14784-400, SP, Brazil; aaguiardefreitas@gmail.com (A.J.A.d.F.); mbertagnav@gmail.com (M.B.V.); stephaniecalfa@outlook.com (S.C.); rhafaela-lima@hotmail.com (R.L.C.); 2 Barretos Cancer Hospital, Barretos 14784-400, SP, Brazil; vinids@gmail.com (V.D.d.S.); crispadua10@gmail.com (C.d.P.S.)

**Keywords:** breast cancer, CNS metastases, immune biomarkers, gene expression, NanoString, personalized oncology

## Abstract

Breast cancer is a leading cause of central nervous system (CNS) metastases in women, often associated with poor prognosis and limited therapeutic options. However, molecular differences between primary tumors and CNS metastases remain underexplored. We aimed to characterize transcriptomic differences between primary breast tumors and matched CNS metastases and identify immune-related biomarkers associated with metastatic progression and patient outcomes. Transcriptomic profiling was based on 11 matched FFPE sample pairs (primary tumor and CNS metastasis). Paired formalin-fixed paraffin-embedded (FFPE) samples from primary tumors (T1) and CNS metastases (T2) were analyzed using the NanoString nCounter^®^ platform and the PanCancer IO 360™ Gene Expression Panel. Differential gene expression, Z-score transformation, and heatmap visualization were performed in R. In silico survival analyses for overall survival (OS) and recurrence-free survival (RFS) were conducted using publicly available TCGA and GEO datasets. Forty-five genes were significantly differentially expressed between the T1 and T2 samples. Immune-related genes such as CXCL9, IL7R, CD79A, and CTSW showed consistent downregulation in CNS metastases. High expression of CXCL9 and CD79A was associated with improved OS and RFS, whereas high IL7R and CTSW expression correlated with worse outcomes. These findings indicate immune suppression as a hallmark of CNS colonization. Comparative transcriptomic analysis further underscored the distinct molecular landscapes between primary and metastatic tumors. This study highlights transcriptional signatures associated with breast cancer CNS metastases, emphasizing the role of immune modulation in metastatic progression. The identified genes have potential as prognostic biomarkers and therapeutic targets, supporting the need for site-specific molecular profiling in metastatic breast cancer management.

## 1. Introduction

According to the Global Cancer Observatory (GLOBOCAN), breast cancer was the most frequently diagnosed cancer in women worldwide in 2022, accounting for 23.8% of all new cancer cases and 15.4% of all cancer-related deaths [1]. In Brazil, data from the National Cancer Institute (INCA) indicate a similar pattern, with 73,610 estimated new breast cancer cases for 2023 and 17,825 recorded deaths in 2020 [2].

Breast cancer is a heterogeneous disease driven by a wide array of genetic and molecular alterations, resulting in substantial clinical and biological variability [3,4,5]. During disease progression, malignant cells can disseminate through lymphatic or hematogenous routes, establishing metastases in distant organs [6,7]. This metastatic capability is associated with genomic instability and the ability of tumor cells to remodel their microenvironment to favor colonization [6]. The molecular underpinnings of metastasis include clonal selection, epithelial–mesenchymal transition (EMT), and immune system modulation, all of which may differ depending on the metastatic site [8].

The central nervous system (CNS) is a notable site for breast cancer metastases, with a reported incidence of 10–16% [9] in clinical series and up to 30% in autopsy studies [10,11]. Breast cancer is the second most common cause of CNS metastases after lung cancer [12]. Without treatment, the average survival after CNS involvement is approximately four weeks. Even with interventions such as radiotherapy or neurosurgery, only about 20% of patients survive beyond one year [13,14,15].

Recent studies demonstrate that there might be a change in the biological pattern of the disease in breast cancer metastasis compared to the primary tumor [16,17,18]. Changes in the expression of Human Epidermal Growth Factor Receptor 2 (HER2), Estrogen Receptor (ER), and/or Progesterone Receptor (PR) status have been described [19,20,21]. In addition, other authors have identified changes in the signaling pathway of recognized markers (EGFR, RAS, PIK3CA, HER2) in breast cancer brain metastases [22]. However, due to the difficulty of accessing biological material in this metastasis location, in addition to the scarcity of studies on this scenario, not much is known about the molecular events that lead to CNS metastasis [23].

Treatment options for metastatic breast cancer are limited and include surgical resection, radiation therapy to the entire skull, chemotherapy, and targeted therapies [24,25]. CNS metastases develop in a specific microenvironment, with highly evolved adaptive manifestations, thus providing a unique opportunity to investigate the influence of the tumor microenvironment on the metastatic process [26,27]. Studies show that for micrometastases to be able to colonize the neural niche, originally hostile, it is necessary to change the microenvironment, which essentially involves the transformation of the glial compartment into a tumor support mechanism [28,29,30].

Due to the uncertainty regarding the origin of multiple genetic abnormalities and their potential clinical utility, molecular targets have shown an important role in clarifying tumor biology [31,32]. A recent study demonstrated intratumoral heterogeneity in metastatic capacity by analyzing gene expression in phylogenies [33]. Another study, with an animal model of metastatic breast cancer, showed a difference in the expression of several genes when comparing metastatic tissue with primary tumors, confirming the fact that intratumoral heterogeneity plays an important role in the selection of clones with metastatic potential [34] and as a target for therapy.

Taken together, these observations underscore the urgent need for a deeper understanding of the tumor microenvironment and the molecular features of CNS metastases in breast cancer. Here we describe transcriptomic profiles associated with CNS metastasis in advanced breast cancer and allow the creation of a standard for clinical trials that personalize the treatment, seeking greater efficacy and better clinical results. In this study, we characterize the transcriptomic profiles associated with CNS dissemination in advanced breast cancer, aiming to identify potential biomarkers and guide the development of personalized treatment strategies to improve clinical outcomes.

## 2. Results

### 2.1. Identification of Differentially Expressed Genes

The analysis revealed 45 genes with significant differential expression between primary tumors (T1) and CNS metastases (T2). These genes were identified based on logFC (log fold-change) values, which indicate the magnitude of gene expression alterations, and statistically significant *p*-values. Among the most significantly altered genes were CXCL9, IL7R, CD79A, and CTSW. For instance, CXCL9 exhibited a significant reduction (logFC = −4.26, *p* = 0.000001), with a Z-score indicating a marked decrease in expression in CNS metastases compared to primary tumors (Table 1).

### 2.2. Categorization by Z-Scores

**Figure 1:**
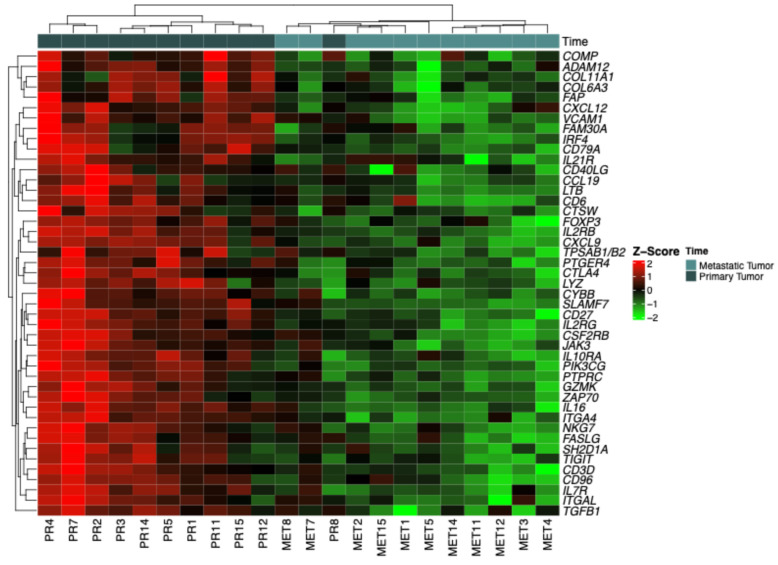
Heatmap representing differential gene expression between primary tumors (T1) and CNS metastases (T2).

The heatmap (Figure 1) presents gene expression data standardized using Z-scores, allowing for a relative comparison between primary tumors (T1) and CNS metastases (T2). The heatmap visually demonstrates how gene expression varies between primary tumors (T1) and CNS metastases (T2) in breast cancer patients. Shades of red represent upregulation (higher expression), green indicates downregulation (lower expression), and neutral tones reflect expression near the mean (Z-score ≈ 0). Genes with intense red coloration have higher expression levels, suggesting activation or increased transcriptional activity in these samples. Conversely, genes with intense green coloration show decreased expression, indicating suppression or downregulation in the context of metastasis compared to the primary tumor. This visualization highlights distinct transcriptional patterns associated with CNS metastasis in breast cancer patients.

### 2.3. In Silico Analysis of Overall Survival and Recurrence-Free Survival

**Figure 2:**
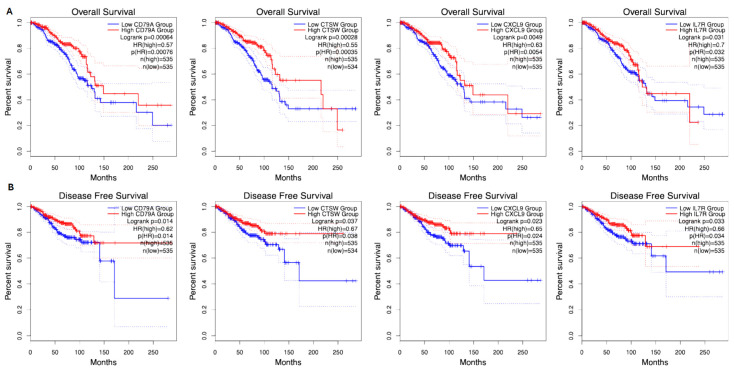
In silico analysis of (**A**) overall survival and (**B**) recurrence-free survival.

Survival analyses using publicly available datasets evaluated the impact of gene expression levels on overall survival (OS) and recurrence-free survival (RFS). Using publicly available datasets, patients were filtered specifically for breast cancer, and survival analyses were conducted for all identified genes. Among all the analyzed genes, CXCL9, IL7R, CD79A, and CTSW were the most significantly associated with both OS and RFS (Figure 2).

### 2.4. Overall Survival (OS)

Figure 2A shows the Kaplan–Meier survival curves for overall survival, highlighting the impact of gene expression levels on patient prognosis. Significant genes such as CXCL9, IL7R, CD79A, and CTSW were analyzed, and their corresponding *p*-values and hazard ratios (HRs) are detailed: CXCL9: *p*-value = 0.0001, HR = 0.68. Patients with high CXCL9 expression (red curve) have a lower risk of death, indicated by an HR < 1. IL7R: *p*-value = 0.002, HR = 1.45. High expression (red curve) is associated with an increased risk of death, as reflected by an HR > 1. CD79A: *p*-value = 0.003, HR = 0.74. High expression correlates with better survival outcomes, with an HR < 1 indicating reduced mortality risk. CTSW: *p*-value = 0.005, HR = 1.62. High expression (red curve) corresponds to a higher mortality risk, as shown by HR > 1. These survival curves illustrate the differential impact of gene expression on overall survival, with red and blue curves representing high- and low-expression groups, respectively.

### 2.5. Recurrence-Free Survival (RFS)

Figure 2B depicts the Kaplan–Meier curves for recurrence-free survival, demonstrating how gene expression levels affect cancer recurrence: CXCL9: *p*-value = 0.0002, HR = 0.72. High expression (red curve) is linked to a reduced risk of recurrence, reflected by an HR < 1. IL7R: *p*-value = 0.004, HR = 1.38. Patients with high IL7R expression (red curve) face a higher risk of recurrence, as indicated by HR > 1. CD79A: *p*-value = 0.007, HR = 0.76. High expression (red curve) correlates with a lower risk of recurrence, suggesting protective effects with HR < 1. CTSW: *p*-value = 0.009, HR = 1.55. High expression (red curve) is associated with an increased likelihood of recurrence, as HR > 1 denotes a higher recurrence risk.

### 2.6. Gene Expression Comparison: Primary Tumor (T1) vs. Metastasis (T2)

**Figure 3:**
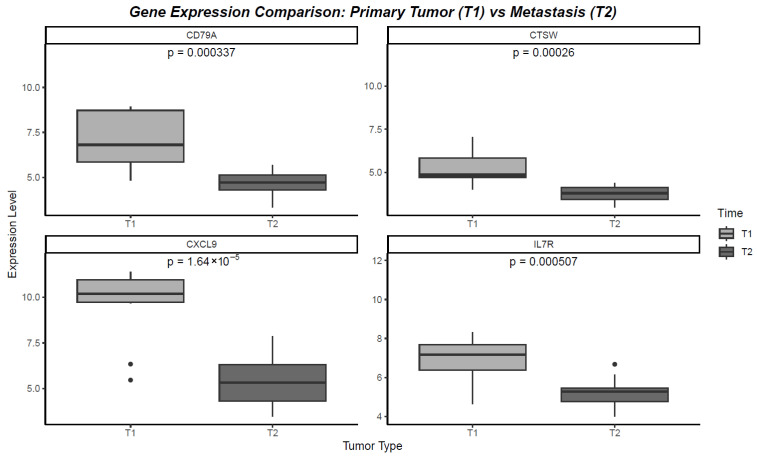
Gene expression comparison: primary tumor (T1) vs. metastasis (T2).

The genes CXCL9, IL7R, CD79A, and CTSW were selected for their significant associations with overall survival (OS) and recurrence-free survival (RFS) in breast cancer patients. Analyzing their expression levels in paired samples of primary tumors (T1) and CNS metastases (T2) revealed notable changes that reflect their roles in tumor progression and adaptation. Upon analyzing their expression in paired primary tumors (T1) and CNS metastases (T2), all four genes exhibited a significant decrease in expression in the metastatic setting. Specifically, CXCL9 showed a marked reduction in expression from T1 to T2, with a significant *p*-value of *p* = 1.64 × 10^−5^, indicating a loss of immune-related function that may contribute to immune evasion in metastases. IL7R also displayed a significant decline in expression (*p* = 0.000507), suggesting that the diminished immune signaling pathways may facilitate tumor progression in the CNS environment. CD79A was similarly downregulated in T2 compared to T1 (*p* = 0.000337), reflecting a decrease in B-cell activity, which could weaken the immune surveillance in the metastatic context. Finally, CTSW experienced a notable reduction in expression between T1 and T2 (*p* = 0.00026), suggesting that the suppressed immune response in metastases might be linked to decreased tumor suppressive activities. These consistent decreases in gene expression highlight the immune-modulatory changes that occur during metastasis to the CNS, emphasizing the potential of these genes as critical biomarkers and therapeutic targets in breast cancer (Figure 3).

### 2.7. Functional Enrichment Analysis of Immune-Related Genes

To explore the biological relevance of the key immune-related genes identified as differentially expressed between primary breast tumors and CNS metastases, we performed a pathway enrichment analysis using curated gene sets from KEGG, Reactome, and other canonical databases. The analysis revealed statistically significant enrichment (FDR < 0.05) in multiple immune-related and cell signaling pathways, particularly those involving B-cell- and cytokine-receptor-mediated signaling. The top enriched pathways are summarized in Table 2. Notably, CD79A and IL7R were involved in the primary immunodeficiency pathway (FDR = 1.43 × 10^−3^), which represents essential disruptions in adaptive immune cell development and function. CXCL9 and IL7R also contributed to the enrichment of the cytokine–cytokine receptor interaction pathway (FDR = 0.034), suggesting impaired immune communication and recruitment within the CNS metastatic niche. Other enriched pathways involving CD79A included B-cell activation, BCR signaling, and B-cell receptor signaling, supporting the role of altered humoral immunity. IL7R was also enriched in Interleukin-7 signaling, hematopoietic cell lineage, and FoxO signaling, pointing to disturbed lymphocyte homeostasis. CXCL9 appeared in CXCR3-mediated and IL-23-mediated signaling events, pathways known for T-cell trafficking and inflammation. Finally, CTSW showed enrichment in the lysosome and apoptosis sets, both linked to cytotoxic granule function and immune-mediated tumor clearance. These findings suggest that the downregulation of these genes in CNS metastases is not random, but functionally clustered in key immunological processes, contributing to the immune-evasive phenotype of breast cancer brain metastases.

## 3. Discussion

The results of this study contribute significantly to understanding the gene expression profile in breast cancer metastases to the central nervous system (CNS), an area of great importance due to the high mortality associated with these metastases [12]. We observed significant differences in gene expression between primary tumors and CNS metastases, suggesting specific cellular adaptations for colonization in the neural environment. Genes such as CXCL9 [35] and IL7R [36] showed marked decreases in metastases, which may reflect immunological modulation facilitating immune evasion. In the literature, elevated expression of CXCL9 [37] and IL7R [38] was related to an improved breast cancer prognosis. However, for CTSW and CD79A, there is no currently consistent evidence in the literature regarding their specific differential expression in the CNS. A study using machine learning identified CTSW as a prognostic marker for pancreatic cancer, whose decreased expression was related to a worse survival rate [39]. CD79A was used as a B lymphocyte infiltration prognostic marker in oral squamous cell carcinoma [40].

These findings reinforce the role of the tumor microenvironment in metastatic progression. Previous studies have shown that, for micrometastases to thrive in the CNS, a transformation of the glial environment is required to support tumor growth [41,42]. The modulation of immune-related genes observed in metastases in this study aligns with literature emphasizing that the cerebral microenvironment becomes a facilitator for tumor growth [28,43].

Additionally, the impact of gene expression levels on overall survival (OS) and recurrence-free survival (RFS) highlights the potential prognostic value of these markers.

The results also show that primary tumors and their CNS metastases have distinct molecular profiles, indicating that targeted therapies based on the primary tumor’s profile may not be fully effective for metastases. This underscores the need for personalized approaches and specific biomarker analyses for each metastatic site [44].

This study has some important limitations. First, the sample size is relatively small, which may limit the generalizability of the findings. Additionally, the genetic and phenotypic diversity of patients was not extensively analyzed, which could impact gene expression data interpretation and the applicability of findings to broader populations. Another point is that, due to the retrospective nature of the study, variability in sample collection and preservation conditions may have introduced bias in the results. Finally, focusing on specific genes and the lack of additional functional analyses limit the full understanding of the biological mechanisms underlying tumor cell adaptations to the CNS microenvironment.

Despite its limitations, this study offers several strengths that significantly enhance the current understanding of breast cancer metastases to the central nervous system. The inclusion of paired samples from both primary tumors and CNS metastases—a rare and clinically valuable dataset—adds substantial robustness and translational relevance. The use of high-resolution gene expression profiling enabled the identification of distinct transcriptional alterations, particularly involving immune-regulatory pathways.

Importantly, the focus on immune-related genes implicated in tumor surveillance adds prognostic depth to the analysis and reveals potential therapeutic targets. The comparative approach between primary tumors and matched CNS metastases further illuminates key molecular adaptations that occur during neural colonization and tumor progression.

The consistent downregulation of immune-related genes such as CXCL9, IL7R, CD79A, and CTSW in CNS metastases underscores their potential role not only as biomarkers of immune evasion but also as possible therapeutic targets. CXCL9 and CD79A have been associated with favorable prognosis and are involved in T-cell and B-cell recruitment, respectively, suggesting a role in immune surveillance. Conversely, IL7R and CTSW, while associated with worse outcomes in primary tumors, may represent context-dependent immune modulators whose suppression in the CNS could reflect immune exclusion or microenvironmental adaptation. These findings highlight the need for site-specific molecular profiling to guide therapeutic strategies and suggest that immune pathways altered in CNS lesions may provide new opportunities for personalized immunotherapeutic interventions, particularly in patients with brain metastases from breast cancer.

Although high expression of IL7R and CTSW was associated with worse prognosis in survival analyses from publicly available datasets, both genes were found to be significantly downregulated in CNS metastases compared to matched primary tumors. This apparent contradiction can be explained by the differences in the biological context of the datasets used. The survival analyses were conducted using unpaired cohorts, primarily composed of primary breast tumors, whereas the gene expression comparison in our study was based on paired samples from primary tumors and CNS metastases. The observed downregulation of IL7R and CTSW in the metastatic setting likely reflects local immune escape mechanisms or microenvironmental pressures that differ from those in the primary tumor. These genes may therefore play context-dependent roles, promoting immune resistance in primary tumors when overexpressed, but being selectively suppressed in the CNS due to the immunologically distinct environment of the brain. This interpretation underscores the complexity of immune-related gene behavior in different metastatic niches and highlights the importance of site-specific molecular profiling in understanding disease progression.

## 4. Materials and Methods

### 4.1. Sample Collection and RNA Extraction

Samples were obtained from the archives of pathological anatomy of the Barretos Cancer Hospital Pathology Department from female patients who were diagnosed with breast cancer and CNS metastasis. Eleven FFPE paired samples of primary tumors (T1) and CNS metastases (T2) were collected. FFPE tissues were macrodissected under RNase-free conditions prior to RNA extraction using the QIAsymphony platform. After RNA extraction, concentration ranged from 50 to 250 ng/µL with Qubit-confirmed quantification. Quality assessment with NanoDrop A260/280 >1.8 (Thermo Fisher Scientific, Waltham, MA, USA) met NanoString quality criteria.

### 4.2. Gene Expression Analysis Using NanoString Technology

Gene expression profiling was performed using the NanoString nCounter^®^ Analysis System (NanoString Technologies, Inc., Seattle, WA, USA.), which provides a highly reproducible and amplification-free digital readout of mRNA abundance. We employed the PanCancer IO 360™ Gene Expression Panel, which includes 770 genes related to tumor biology, the tumor microenvironment, and immune regulation. Total RNA extracted from FFPE samples was hybridized to custom barcoded probe pairs, consisting of reporter and capture probes specific to each target gene. Hybridization was carried out at 65 °C overnight (21 h), in accordance with the manufacturer’s instructions, ensuring optimal hybrid formation. Following hybridization, the samples were processed using the nCounter Prep Station for automated purification and immobilization on a cartridge, followed by scanning with the nCounter Digital Analyzer, which generates direct counts of hybridized transcripts. The raw data were analyzed using nSolver™ Analysis Software, version 4.0 (NanoString Technologies, Seattle, WA, USA), which included several quality control steps: imaging QC, binding density evaluation, positive and negative control assessment, and CodeSet content validation. Data normalization was performed using the geometric mean of the validated housekeeping genes included in the panel. All samples were processed in a single batch, thereby minimizing technical variation and eliminating the need for batch effect correction.

### 4.3. Identification of Differentially Expressed Genes

Differential gene expression analysis between T1 and T2 was performed using the NanostringNorm package in R version 4.2.2. Genes with significant differences in expression were identified based on log fold-change (logFC) and *p*-values. The significant genes were those with an adjusted *p*-value < 0.05 and a logFC threshold defined according to predefined criteria for clinical relevance.

### 4.4. Z-Score Categorization and Heatmap Visualization

Gene expression data were standardized into Z-scores to compare the relative expression levels of each gene between samples. Heatmaps were generated using the ComplexHeatmap package in R version 4.2.2, displaying expression levels with a red to green color gradient. Red indicated upregulation (high expression) and green indicated downregulation (low expression), helping to visualize expression patterns in primary tumors and metastases.

### 4.5. Survival Analysis

In silico survival analysis was conducted using publicly available datasets from The Cancer Genome Atlas (TCGA) and Gene Expression Omnibus (GEO). Overall survival (OS) and recurrence-free survival (RFS) were assessed for each gene using Kaplan–Meier analysis and Cox proportional hazards models. Patients were stratified based on high and low levels of gene expression, and hazard ratios (HRs) were calculated to determine the impact of gene expression on survival outcomes.

## 5. Conclusions

Our findings highlight a consistent downregulation of immune-modulatory genes—including CXCL9, IL7R, CD79A, and CTSW—in CNS metastases. This suggests that immune suppression plays a critical role in facilitating tumor adaptation to the brain microenvironment. These genes demonstrated prognostic value in in silico survival analyses and may serve as biomarkers for clinical stratification or therapeutic intervention.

Together, these results emphasize the need for site-specific molecular profiling, as relying solely on the primary tumor’s molecular landscape may overlook critical alterations in metastatic lesions. Tailoring treatment strategies based on the biology of the metastatic site may ultimately improve outcomes for patients with breast cancer brain metastases.

## Figures and Tables

**Table 1:** Genes differentially expressed between primary tumors (T1) and CNS metastases (T2).

Genes	Log Fold-Change	*p*-Value	Protein Function
*CXCL9*	−4.26	0.000001	Chemotactic for T-cells
*IRF4*	−2.33	0.000002	Interferon regulation
*IL2RB*	−2.10	0.000003	T-cell-mediated immune response
*IL16*	−1.78	0.000005	Modulator of T-cell activation
*IL2RG*	−1.63	0.000006	Signaling component of interleukin receptors
*CD27*	−2.18	0.000006	Maintenance of T-cell immunity
*ITGA4*	−1.41	0.000009	Cell surface adhesion and signaling
*CXCL12*	−1.66	0.000141	Signaling receptor and chemokine activity
*COMP*	−2.61	0.000153	Extracellular matrix protein
*COL6A3*	−2.32	0.000167	Binding to extracellular matrix
*CD79A*	−2.40	0.000376	B-cell antigen component
*VCAM1*	−1.59	0.000391	Leukocyte–endothelial cell adhesion
*SLAMF7*	−1.54	0.000491	Protein binding activity
*GZMK*	−1.58	0.000553	Serine proteases
*LTB*	−1.99	0.000568	Inflammatory response system
*JAK3*	−1.54	0.000621	Cytokine receptor-mediated
*ADAM12*	−1.55	0.000646	Cell–matrix interactions
*FASLG*	−1.45	0.000670	Induction of apoptosis
*CTSW*	−1.52	0.000736	Regulation of T-cell cytolytic activity
*CSF2RB*	−1.37	0.000839	Interleukin-3 receptor activity
*FAM30A*	−1.58	0.000894	LncRNA class
*CD3D*	−1.72	0.000904	T-cell development and signal transduction
*ZAP70*	−1.44	0.000112	T-cell development and lymphocyte activation
*PIK3CG*	−1.20	0.000117	Cell growth, survival, proliferation, and motility
*FAP*	−1.17	0.000161	Epithelial–mesenchymal interactions
*TPSAB1/B2*	−2.44	0.000170	Tryptases
*IL7R*	−1.68	0.000189	Lymphocyte development
*COL11A1*	−2,00	0.000253	Extracellular matrix structural constituent
*CTLA4*	−1.39	0.000337	Inhibitory signal to T-cells
*LYZ*	−2.03	0.000357	Antitumor immune activity
*PTPRC*	−1.40	0.000365	Cell growth, differentiation, and mitosis
*CD6*	−1.20	0.000388	Binding site for adhesion molecules
*NKG7*	−1.84	0.000417	Regulates cytotoxic granule exocytosis
*PTGER4*	−1.04	0.000435	Activates T-cell factor signaling
*CCL19*	−1.96	0.000465	Immunoregulatory and inflammatory processes
*TIGIT*	−1.45	0.000497	Signaling receptor binding
*CD96*	−1.10	0.000519	Adhesive interactions of activated T- and NK cells
*CYBB*	−1.43	0.000712	Microbicidal oxidase system of phagocytes
*FOXP3*	−0.99	0.000725	DNA-binding transcription factor activity
*CD40LG*	−1.11	0.000758	Regulates B-cell function
*TGFB1*	−0.82	0.000778	Regulates gene expression
*IL21R*	−1.20	0.000835	Proliferation and differentiation of immune cells
*ITGAL*	−1.29	0.000900	Leukocyte intercellular adhesion
*IL10RA*	−1.09	0.000990	Related to interferon receptors
*SH2D1A*	−1.29	0.016551	Stimulation of T- and B-cells

**Table 2:** Pathway enrichment analysis of selected immune-related genes.

Gene Set	Ratio of Proteins	Proteins in Pathway	Genes From Network	*p*-Value	FDR	Involved Genes
Primary immunodeficiency (KEGG)	0.0032	38	2	5.95 × 10^−5^	0.00143	*CD79A, IL7R*
Cytokine–cytokine receptor interaction (KEGG)	0.0245	295	2	3.48 × 10^−3^	0.034	*CXCL9, IL7R*
ctcf: first multivalent nuclear factor (BioCarta)	0.0018	22	1	7.29 × 10^−3^	0.034	*CD79A*
Interleukin-7 signaling (Reactome)	0.0018	22	1	7.29 × 10^−3^	0.034	*IL7R*
BCR signaling pathway (BioCarta)	0.0022	26	1	8.61 × 10^−3^	0.034	*CD79A*
CXCR3-mediated signaling events (NCI)	0.0029	35	1	0.012	0.034	*CXCL9*
IL-23-mediated signaling events (NCI)	0.0031	37	1	0.012	0.034	*CXCL9*
B-cell activation (PID)	0.0039	47	1	0.016	0.034	*CD79A*
BCR signaling pathway (NCI)	0.0054	65	1	0.021	0.034	*CD79A*
B-cell receptor signaling pathway (KEGG)	0.0068	82	1	0.027	0.034	*CD79A*
Hematopoietic cell lineage (KEGG)	0.0082	99	1	0.032	0.034	*IL7R*
Viral protein interaction with cytokine and cytokine receptor (KEGG)	0.0083	100	1	0.033	0.034	*CXCL9*
Toll-like receptor signaling pathway (KEGG)	0.0086	104	1	0.034	0.034	*CXCL9*
Clathrin-mediated endocytosis (Reactome)	0.0103	124	1	0.041	0.041	*IL7R*
Lysosome (KEGG)	0.0106	128	1	0.042	0.042	*CTSW*
FoxO signaling pathway (KEGG)	0.0109	131	1	0.043	0.043	*IL7R*
Apoptosis (KEGG)	0.0113	136	1	0.044	0.044	*CTSW*

## Data Availability

The data presented in this study are available on request from the corresponding author. The data are not publicly available due to restrictions related to patient confidentiality and institutional data use policies.
